# Diagnosis and treatment of Ascarial pancreatitis: a case report and literature review

**DOI:** 10.1093/omcr/omaf135

**Published:** 2025-08-20

**Authors:** Hongyu He, Hao Guo, Zhongtao Li, Shu Wang, GuoJun Zhou, Zhi Liu, Jianyu Chen, Zhengwei Leng, Liang Xie

**Affiliations:** Department of General Surgery (Wenhua Road Campus), The Affiliated Hospital of North Sichuan Medical College, No. 63, Wenhua Road, Shunqing District, Nanchong, Sichuan 637000, China; Institute of Hepatobiliary Pancreatic and Intestinal Diseases, Shunqing Campus, North Sichuan Medical College, No. 234, Fujiang Road, Shunqing District, Nanchong, Sichuan 637000, China; Department of General Surgery (Wenhua Road Campus), The Affiliated Hospital of North Sichuan Medical College, No. 63, Wenhua Road, Shunqing District, Nanchong, Sichuan 637000, China; Institute of Hepatobiliary Pancreatic and Intestinal Diseases, Shunqing Campus, North Sichuan Medical College, No. 234, Fujiang Road, Shunqing District, Nanchong, Sichuan 637000, China; Department of General Surgery (Wenhua Road Campus), The Affiliated Hospital of North Sichuan Medical College, No. 63, Wenhua Road, Shunqing District, Nanchong, Sichuan 637000, China; Institute of Hepatobiliary Pancreatic and Intestinal Diseases, Shunqing Campus, North Sichuan Medical College, No. 234, Fujiang Road, Shunqing District, Nanchong, Sichuan 637000, China; Department of Urology Surgery, Affiliated Hospital of North Sichuan Medical College (Maoyuan South Road Campus), No. 1, Maoyuan South Road, Shunqing District, Nanchong, Sichuan 637000, China; Institute of Hepatobiliary Pancreatic and Intestinal Diseases, Shunqing Campus, North Sichuan Medical College, No. 234, Fujiang Road, Shunqing District, Nanchong, Sichuan 637000, China; Department of Hepatobiliary Surgery,Affiliated Hospital of North Sichuan Medical College (Maoyuan South Road Campus), No. 1, Maoyuan South Road, Shunqing District, Nanchong, Sichuan 637000, China; Institute of Hepatobiliary Pancreatic and Intestinal Diseases, Shunqing Campus, North Sichuan Medical College, No. 234, Fujiang Road, Shunqing District, Nanchong, Sichuan 637000, China; Department of Hepatobiliary Surgery,Affiliated Hospital of North Sichuan Medical College (Maoyuan South Road Campus), No. 1, Maoyuan South Road, Shunqing District, Nanchong, Sichuan 637000, China; Institute of Hepatobiliary Pancreatic and Intestinal Diseases, Shunqing Campus, North Sichuan Medical College, No. 234, Fujiang Road, Shunqing District, Nanchong, Sichuan 637000, China; Department of Hepatobiliary Surgery,Affiliated Hospital of North Sichuan Medical College (Maoyuan South Road Campus), No. 1, Maoyuan South Road, Shunqing District, Nanchong, Sichuan 637000, China; Institute of Hepatobiliary Pancreatic and Intestinal Diseases, Shunqing Campus, North Sichuan Medical College, No. 234, Fujiang Road, Shunqing District, Nanchong, Sichuan 637000, China; Department of Hepatobiliary Surgery,Affiliated Hospital of North Sichuan Medical College (Maoyuan South Road Campus), No. 1, Maoyuan South Road, Shunqing District, Nanchong, Sichuan 637000, China; Department of General Surgery (Wenhua Road Campus), The Affiliated Hospital of North Sichuan Medical College, No. 63, Wenhua Road, Shunqing District, Nanchong, Sichuan 637000, China; Institute of Hepatobiliary Pancreatic and Intestinal Diseases, Shunqing Campus, North Sichuan Medical College, No. 234, Fujiang Road, Shunqing District, Nanchong, Sichuan 637000, China

**Keywords:** acute pancreatitis, Ascaris lumbricoides worms, ultrasonic, ERCP, diagnosis

## Abstract

Background: The prevalence of diseases caused by parasites has decreased due to improved sanitary conditions. Acute pancreatitis caused by parasites, especially Ascaris lumbricoides worms, is relatively rare and difficult to diagnose. In some cases, it may even be misdiagnosed as idiopathic acute pancreatitis due to the difficulty of identifying the underlying cause. Research indicates that about 1.4 billion people worldwide are infected with Ascaris lumbricoides worms, and pancreatitis caused by roundworms accounts for only 5.50% of the total cases. Therefore, it is imperative to gain a comprehensive understanding of the pathological process, diagnosis, and treatment of pancreatitis caused by Ascaris lumbricoides worms. Case Presentation: We describe a case of Ascariasis-induced pancreatitis in an 82-year-old woman who was admitted to our emergency department with persistent abdominal pain, nausea, and vomiting for 6 h. Abdominal magnetic resonance imaging and magnetic resonance cholangiopancreatography revealed abnormal tubular signals in the common bile duct segment, accompanied by dilation of the hilar and common bile ducts. Consequently, a diagnosis of Ascariasis-induced pancreatitis was made. Result: Ascariasis-induced pancreatitis was detected in individuals of all age groups and genders. Most cases occurred in Asia (43 cases, 66.15%) and Europe (8 cases, 12.3%). The most common symptoms were abdominal pain and fever. The diagnosis was primarily by ultrasound examination (43.3%) and endoscopic procedures. Regarding treatment, 76.7% of the patients received antiparasitic drugs, while 85.45% underwent endoscopic procedures to directly remove the Ascaris worms. In our case, the patient underwent laparoscopic procedures to remove a 20 cm-long ascaris worms and alleviate symptoms. Conclusion: Ascariasis-induced pancreatitis is more commonly detected among Asians, being more frequent with adult females. The clinical symptoms are often atypical compared to those of pancreatitis caused by other etiologies. In cases of acute pancreatitis resulting from biliary ascariasis, it is recommended that clinicians employ a combination of imaging modalities to support the diagnostic process. The literature indicates that endoscopic retrograde cholangiopancreatography (ERCP) has been the primary treatment approach in the majority of reported cases. In recent years, laparoscopic surgery has been found to be associated with faster recovery and reduced trauma. In complex cases involving severe cholecystitis or intrahepatic biliary ascariasis, laparoscopy offers distinct and irreplaceable benefits.

## Introduction

Ascaris worms are the largest parasitic nematodes in the human gastrointestinal tract. In recent years, its infection rate has decreased due to personal and environmental hygiene improvements. However, it remains the most common pathogenic parasite among school-age children [1]. Roundworms can migrate within the body, complete their unique life cycle, and can enter the bile ducts from the intestine. This increases the risk of both gallstone formation and roundworm infections in humans. Further blockage of the bile duct or pancreatic duct by roundworms can lead to acute cholecystitis and acute pancreatitis, causing gastrointestinal symptoms. This has always been a major public health issue worldwide.

Acute pancreatitis is an inflammatory disease affecting the pancreas. It is caused by deregulation of pancreatic digestive enzymes, leading to self-digestion of pancreatic tissue. Its common symptoms include severe abdominal pain, nausea, vomiting, fever, and abdominal bloating. The incidence rate of acute pancreatitis is increasing globally year after year. The most common causes are obstruction of the bile duct by gallstones (38%) and alcohol abuse (36%) [2]. Other less frequent causes include metabolic factors (such as hypercalcemia and hypertriglyceridemia), drug-induced factors, and autoimmune factors. A report by Jordan P. Iannuzzi found that the incidence rate of acute pancreatitis is on the rise in most regions. The average annual percent change (AAPC) varies by continent, with North America at 3.67%, Europe at 2.77%, and Asia at 0.28% [3]. Regarding its etiology, a definite cause is generally identified in approximately 75% to 85% of patients. Among the causes, parasitic factors are gradually decreasing due to personal and environmental hygiene improvements. As the most common parasitic pathogen worldwide, Ascaris worm infections affect approximately one billion people globally. Ascaris lumbricoides worms are mainly prevalent in Southeast Asia, Africa, China, and Latin America [4]. Infection with Ascaris lumbricoides worms is mainly caused by exposure of the host to environments (water sources, soil, and raw food) that are contaminated with roundworms [5]. According to a study by Khuroo MS, ascariasis-induced pancreatitis accounts for only 5.50% of cases [6]. In another study by the same author, Ascaris lumbricoides worms were observed in the biliary tract of 5 out of 1104 patients undergoing abdominal ultrasound examination [7]. Given the relatively rare infection rates of Ascaris lumbricoides worms, they are rarely considered as a potential cause of pancreatitis. Ultrasound, despite its high sensitivity and widespread use, has limitations. Unpredictable errors can occur due to variations in equipment across hospitals and patient factors during the examination. For instance, in our case report, the initial ultrasound failed to detect any abnormalities. Consequently, some doctors may misdiagnose the patient with idiopathic pancreatitis and initiate conservative treatment. Consequently, they may be unprepared for a rapid decline in the patient's condition. This article aims to address this challenge by providing a retrospective analysis of all literature on ascariasis-induced pancreatitis published between 2003 and 2023. In addition, we will present a case study of a patient with pancreatitis caused by Ascaris lumbricoides worms infection.

## Case presentation

### Case report

This case presents an 82-year-old elderly female who presented to our emergency department with persistent abdominal pain for 6 h as the main complaint. The patient also manifested symptoms of nausea and vomiting. The vomitus consisted of gastric fluid and stomach contents. The pain slightly dissipated after vomiting, and it was also associated with heartburn, acid reflux, and dizziness. The patient had no signs of abdominal distension, diarrhea, melena, or hematemesis. Emergency laboratory investigations revealed amylase levels of 3347 U/l, lipase levels of 6090 U/l, and amylase levels of 3367 U/l. Abdominal ultrasound findings revealed high echogenicity filling of the right intrahepatic bile duct, with intrahepatic and extrahepatic bile ducts dilatation, fatty liver, and hepatic cyst. The patient's medical history includes uterine fibroid surgery over 30 years ago and conservative treatment for a right shoulder injury 2 years ago. She has been on long-term oral omeprazole for "gastric pain" and received an unknown oral medication for toothache at a clinic one day before admission. Otherwise, her medical history is unremarkable. Physical examination revealed abdominal tenderness throughout, with greater intensity in the upper abdomen. Based on the abdominal examination, rebound tenderness could be felt throughout the abdomen. No abdominal muscle tension and palpable masses were detected in the abdomen. The liver and spleen were not palpable and Murphy's sign was negative. In addition, no hepatic or renal percussion tenderness, mobility dullness, and percussion pain were detected in both renal regions. At last, there is one additional point to note: the patient's bowel sounds have become weaker.

### Intervention and results

**Figure 1:**
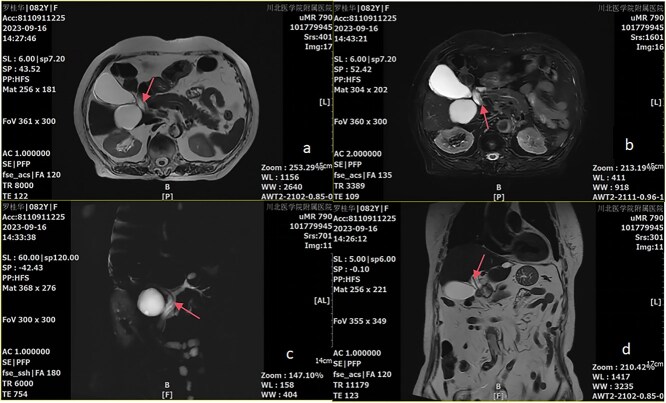
Magnetic resonance T2-weighted imaging (T2WI) (abcd)(red arrows point to the roundworm imaging finding in the common bile duct).

Upon admission, further examinations showed that the white blood cell count was increased to 13.48x10E9/L, the neutrophil percentage was increased to 88.50%, and the absolute neutrophil count increased to 11.94x10E9/L. The lymphocyte percentage was reduced to 8.70%, and the monocyte percentage decreased to 2.80%. Further analysis found that eosinophil percentage was reduced to 0x10E9/L. There were no anomalies in the plasma D-dimer which had a concentration of 1.86Ug/ml. The levels of cardiac enzymes were within normal ranges. The concentration of liver enzymes was elevated. Total bilirubin 18.7 umol/L, direct bilirubin 4.4 umol/L, and indirect bilirubin 14.3 umol/L all remain within normal reference ranges. Abdominal X-ray in the upright position showed no abnormalities. An upper abdominal Computer Tomography (CT) scan revealed cholecystolithiasis in the porta hepatis with mild dilation of the intrahepatic and hepatic ducts. In addition, some cysts were seen in the liver, and high-density nodules in the right anterior lobe of the liver. These findings suggested calcifications or stones. The diagnosis of pancreatitis was confirmed through a combination of CT imaging results and pre-admission laboratory markers specific to the condition. Suspected choledocholithiasis, along with possible biliary obstruction, was identified as the underlying cause of this episode. However, unexpected results from further refined tests prompted a change in course. Abdominal Magnetic Resonance Imaging (MRI) and Magnetic Resonance Cholangiopancreatography (MRCP) were performed for further investigation. These studies revealed a tubular abnormal signal within the common bile duct (choledochus) of an undetermined nature (Fig. 1). Additionally, dilation of the porta hepatis bile duct and the common bile duct was observed.

**Figure 2:**
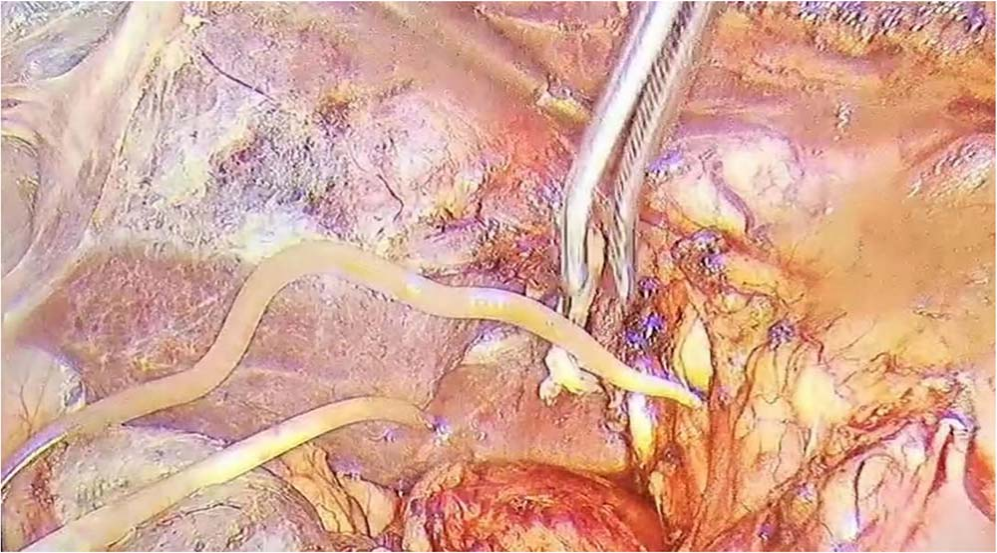
Intraoperative removal of ascaris worms.

Following admission, the patient was put on standard treatment for pancreatitis, including inhibition of pancreatic enzymes, liver protection, acid suppression, anti-inflammatory medication, pain relief, and maintenance of fluid and electrolyte balance. Despite conservative treatment, the patient's symptoms did not improve significantly. In addition, mild jaundice developed, and the patient's abdominal pain worsened. Chills and fever also began to appear. Therefore, a decision was made to intervene further to prevent disease progression. On September 22, 2023, the patient underwent laparoscopic surgery under general anesthesia, which included Laparoscopic common bile duct T-tube drainage, Laparoscopic cholecystectomy, and Abdominal lavage drainage. During the surgery, adhesions were found in the upper right abdomen between the greater omentum, abdominal wall, liver, and gallbladder. The gallbladder was thickened and enlarged, with a wall thickness measuring approximately 6 mm. In addition, intra-gallbladder pressure was elevated. During the postoperative examination of the gallbladder, a stone with a diameter of 0.8 cm was observed. Further, a cystic duct with a diameter of about 3 mm was detected, and no stone was observed it in. The extrahepatic bile ducts were visibly dilated, but the liver appeared normal in color, texture, and morphology. The diameter of the common hepatic and common bile ducts were approximately 1.0 cm and 1.5 cm, respectively. Examination of the common bile duct identified a white worm measuring approximately 20 cm long, which was removed with forceps (Fig. 2). Subsequent endoscopic examination revealed a small amount of fluffy material in the lower segment of the common bile duct. The sphincter of Oddi function was normal, and no other significant abnormalities were identified.

The worms removed during surgery were confirmed to be Ascaris lumbricoides. The patient's symptoms significantly improved following surgery. On the first postoperative day, liver function tests revealed the following bilirubin levels: Total Bilirubin 16.2umol/L, Direct Bilirubin 5.8umol/L, and Indirect Bilirubin 10.4umol/L. In consultation with the Department of Infectious Diseases, fecal parasite microscopy was performed after the patient's condition stabilized. Albendazole 400 mg was then administered orally. Subsequent tests revealed no roundworm eggs in the stool. Additionally, the patient's blood tests showed significant improvement upon re-examination. The patient was subsequently discharged from the hospital.

## Literature review

### Review of the literature

#### Methods

**Figure 3:**
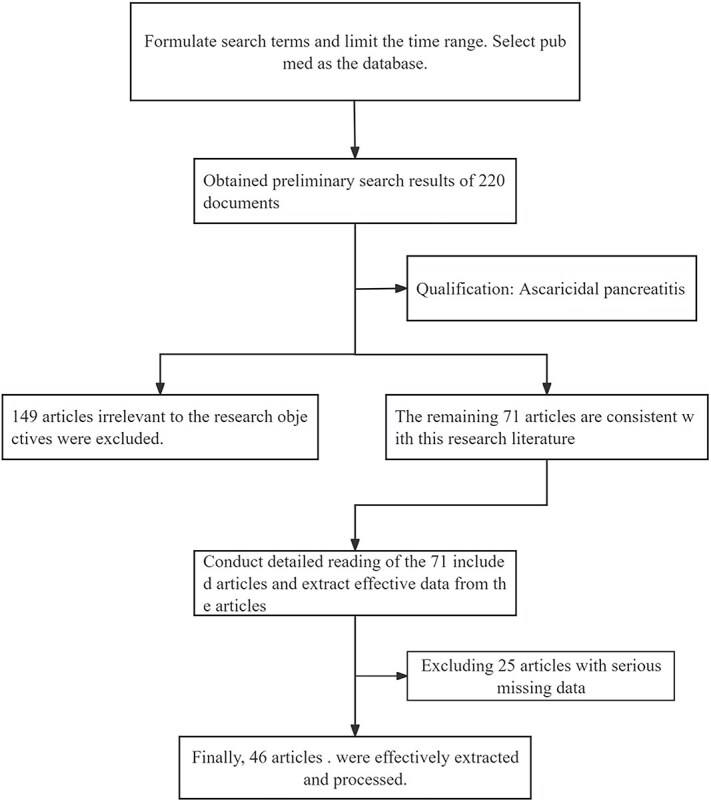
Search, inclusion, elimination process.

A retrospective search was conducted on the PubMed database from 2003 to 2023 to identify relevant case report literature. The following search terms were used: #1 pancreatitis, #2 roundworm OR ascaris lumbricoides OR ascaris. The combination of terms was: #1 AND #2. The search identified 220 studies. Among them, 149 articles were excluded because they did not meet the specified criteria for pancreatitis caused by roundworm, and the remaining 71 articles were included in the final analysis. Screening of the 71 articles led to further identification of 25 articles as they lacked inadequate data for statistical analysis. Finally, data on ascariasis-induced pancreatitis were extracted and analyzed from the remaining 46 articles (Fig. 3).

## Result

Ascaris-induced pancreatitis can occur in patients of all age groups, with a mean age of onset of 32.17 ± 18.38 years (as shown in Table 1). The youngest reported case was a 5-year-old child, while the oldest was 78. Adults account for most cases, with only 14 pediatric patients, including five children under 10. The number of female patients (35 cases) was higher than that of male patients (25 cases).

A significant geographic concentration of cases is observed in Asia, representing about 66.15% (43 cases) of the total reported (as shown in Table 1). Among these, India accounts for the largest share with 26 cases, while Pakistan follows with 7 cases. Europe ranks second in case numbers, with 8 cases (around 12.3%), while reports from other regions are relatively rare. This situation may be related to the global prevalence and distribution of ascariasis [4].

Due to variations in symptom descriptions across different studies, we mainly focused on the three most commonly reported symptoms (as shown in Table 2). Abdominal pain is the most prevalent, with 55 patients (91.7%) presenting with typical left upper quadrant pain. Fever was reported in 17 patients (28.3%), typically occurring after the onset of other symptoms. Vomiting of ascarids occured in eight patients (13.3%), primarily in those with heavy worm burdens.

Several imaging-based diagnostic modalities are employed at the initial presentation, including conventional abdominal ultrasound, endoscopic ultrasound, ERCP, and computed tomography (as shown in Table 2). Conventional ultrasound is used most frequently as the initial diagnostic tool.

**Table 1:** Clinical characteristics of patients.

Case No.	Gender	Age	Clinical symptoms			Country	First diagnostic imaging test	Antiparasitic agent	Surgery	Reference
			Abdominal pain	Vomiting roundworm	Fever					
1	Female	15	Yes	NO	NO	Turkey	Ultrasonic	YES	NO	Korcan Aysun GÖNEN et al. [8]
2	Female	33	NO	NO	NO	India	EUS	YES	NO	Malay Sharma et al. [8]
3	Female	30	NO	NO	NO	India	EUS	YES	NO	Sharma Malay et al. [13]
4	Female	28	Yes	NO	NO	India	ERCP	YES	NO	Malay Sharma et al. [11]
5	Female	36	Yes	NO	YES	China	ERCP	YES	NO	Naveed Khan et al. [14]
6	Female	60	Yes	NO	NO	Bolivia	ERCP	YES	NO	Rosario Ruiz Domı’nguez et al. [15]
7	Male	77	Yes	NO	YES	Croatia	ERCP	YES	NO	Brankica Mijandrusˇic´-Sincic et al. [16]
8	Female	30	Yes	NO	NO	India	Ultrasonic	YES	NO	Donboklang Lynser et al. [15]
9	Female	27	Yes	NO	NO	India	Ultrasonic	NO	NO	Donboklang Lynser et al. [15]
10	Female	26	Yes	NO	NO	India	Ultrasonic	NO	NO	Donboklang Lynser et al. [15]
11	Female	14	Yes	NO	NO	India	Ultrasonic	NO	NO	Donboklang Lynser et al. [15]
12	Male	66	Yes	NO	YES	Korea	ERCP	YES	NO	Kyo-Sang Yoo et al. [17]
13	Male	46	Yes	YES	YES	England	——	YES	NO	Dominic Pimenta et al. [18]
14	Male	59	Yes	NO	NO	Peru	ERCP	YES	NO	Gerly Edson Guzman et al. [17]
15	Female	33	Yes	NO	YES	Thailand	ERCP	YES	NO	Pochamana Phisalprapa et al. [19]
16	Female	73	Yes	NO	NO	Lithuania	ERCP	YES	NO	Michail Klimovskij et al. [20]
17	Male	12	Yes	NO	NO	Pakistan	Ultrasonic	YES	NO	Mahrukh Afreen et al. [18]
18	Male	8	Yes	NO	NO	Pakistan	Ultrasonic	YES	NO	Mahrukh Afreen et al. [19]
19	Male	5	Yes	NO	NO	Pakistan	Ultrasonic	NO	NO	Mahrukh Afreen et al. [19]
20	Male	58	Yes	NO	NO	Spain	——	YES	NO	María Dolores Casado-Maestre et al. [21]
21	Female	78	Yes	YES	YES	Italy	——	YES	whipple procedure	A. Galzerano et al. [22]
22	Male	25	Yes	YES	YES	Pakistan	——	YES	NO	Taimoor Hussain et al. [23]
23	Male	37	Yes	NO	NO	Italy	EUS	YES	NO	Benedetto Mangiavillano et al. [24]
24	Male	62	Yes	NO	NO	Korea	ERCP	NO	NO	Tae Hoon Lee et al. [25]
25	Male	18	Yes	NO	NO	Spain	ERCP	YES	NO	Carter A. Kenamond et al. [26]
26	Female	40	Yes	YES	YES	Mexico	Ultrasonic	YES	Cholecystectomy+exploration of common bile duct incision	Mauricio de la Fuente-Lira et al. [27]
27	Female	40	NO	NO	NO	Spain	Ultrasonic	YES	NO	Lucía Tortajada-Laureiro et al. [28]
28	Male	16	Yes	NO	YES	South Africa	Ultrasonic	NO	NO	JAKE KRIGE et al. [27]
29	Female	—	NO	NO	NO	Columbia	ERCP	NO	NO	E. Zapata et al. [29]
30	Male	36	Yes	NO	YES	Thailand	ERCP	YES	NO	S. Leelakusolvong et al. [30]
31	Male	30	Yes	NO	NO	India	EUS	YES	NO	Piyush Somani et al. [31]
32	Male	24	Yes	YES	YES	India	EUS	YES	NO	Malay Sharma et al. [30]
33	Male	37	Yes	NO	NO	America	Ultrasonic	YES	Cholecystectomy	Gregg Miller et al. [32]
34	Male	11	Yes	YES	NO	India	Ultrasonic	NO	NO	Donboklang Lynser et al. [33]
35	Female	5	Yes	NO	NO	India	Ultrasonic	NO	NO	Donboklang Lynser et al. [33]
36	Female	11	Yes	NO	NO	India	Ultrasonic	NO	NO	Donboklang Lynser et al. [33]
37	Female	5	Yes	NO	NO	India	Ultrasonic	NO	NO	Donboklang Lynser et al. [33]
38	Male	52	Yes	NO	YES	Ethiopia	Ultrasonic	NO	NO	Rodas Temesgen et al. [34]
39	Female	52	Yes	YES	NO	Pakistan	Ultrasonic	NO	NO	Muhammad Azhar et al. [29]
40	Female	35	Yes	NO	YES	India	Ultrasonic	YES	Laparoscopic cholecystectomy	Uptal De et al. [35]
41	Female	31	Yes	NO	NO	India	Ultrasonic	YES	NO	Uptal De et al. [35]
42	Male	32	Yes	NO	YES	India	Ultrasonic	YES	Liver abscess fenestration and drainage + roundworm removal	Uptal De et al. [35]
43	Female	27	Yes	NO	YES	India	Ultrasonic	YES	NO	Uptal De et al. [35]
44	Female	50	Yes	NO	NO	Micronesia	EUS	YES	NO	Morgan Freeman et al. [36]
45	Female	17	Yes	NO	NO	India	EUS	YES	NO	Malay Sharma et al. [35]
46	Male	34	Yes	NO	NO	India	EUS	YES	NO	Malay Sharma et al. [35]
47	Male	26	Yes	NO	NO	India	EUS	YES	NO	Malay Sharma et al. [35]
48	Female	27	Yes	NO	NO	India	EUS	YES	NO	Malay Sharma et al. [35]
49	Female	12	Yes	NO	NO	India	EUS	YES	NO	Malay Sharma et al. [35]
50	Female	10	Yes	YES	NO	Pakistan	CT	YES	NO	Komal Samir et al. [1]
51	Female	43	Yes	NO	NO	India	EUS	YES	whipple procedure	S Arulprakash et al. [37]
52	Male	19	Yes	NO	NO	India	Ultrasonic	NO	NO	Agarwal A et al. [38]
53	Female	56	Yes	NO	NO	China	EUS	YES	NO	Wei Liu et al. [39]
54	Female	32	Yes	NO	YES	India	Ultrasonic	YES	Ultrasound guided liver puncture	Indranil Chakrabarti et al. [40]
55	Female	24	Yes	NO	NO	Nepal	Ultrasonic	YES	NO	Thakur SK et al. [41]
56	Male	8	NO	NO	NO	Pakistan	ERCP	YES	NO	Huma Arshad Cheema et al. [40]
57	Male	21	Yes	NO	NO	America	EUS	YES	NO	Madhusudhan R. Sanaka et al. [42]
58	Female	21	Yes	NO	NO	Turkey	Ultrasonic	YES	Exploratory laparoscopy+intraoperative cholangiography+common bile duct incision for worm removal	Nazim Ag˘aog˘lu et al. [43]
59	Female	34	Yes	NO	NO	The People's Republic of Bangladesh	ERCP	YES	NO	Lee K H et al. [44]
60	Female	24	Yes	NO	YES	India	ERCP	YES	NO	R AGRAWAL et al. [45]

**Table 2:** Summary of clinical characteristics.

Clinical feature				
Feature				Proportion
Age	maximum age	78	average age:32.17 ± 18.38	——
	minimum age	5		
Gender	male	25		41.70%
	female	35		58.30%
Region	Asia	43		66.15%
	Europe	8		12.30%
	North America	3		4.61%
	South America	3		4.61%
	Africa	2		3.08%
	Oceania	1		1.54%
Clinical manifestations	Left upper abdominal pain	55		91.70%
	Fever	17		28.30%
	Vomiting oundworms	8		13.30%
non-pharmacological treatment	ERCP	47		85.45%
	Surgical treatment	‘Whipple’ surgery 2		3.64%
		Cholecystectomy 2		3.64%
		Cholecystectomy + common bile duct incision and exploration 2		3.64%
	Other therapeutic procedures	Liver puncture/window drainage, etc. 2		3.64%

Regarding treatment, 46 patients (76.7%) received anthelmintic drug therapy (as shown in Table 2). The remaining patients underwent non-pharmacological interventions, such as ERCP, Whipple procedure, cholecystectomy, cholecystectomy with common bile duct exploration, and percutaneous drainage. Among these, ERCP was the most commonly used non-drug treatment, with 47 patients (85.45%) having ascarids directly extracted during the ERCP procedure. The other surgical methods mentioned are used much less frequently.

## Disccusion

**Figure 4:**
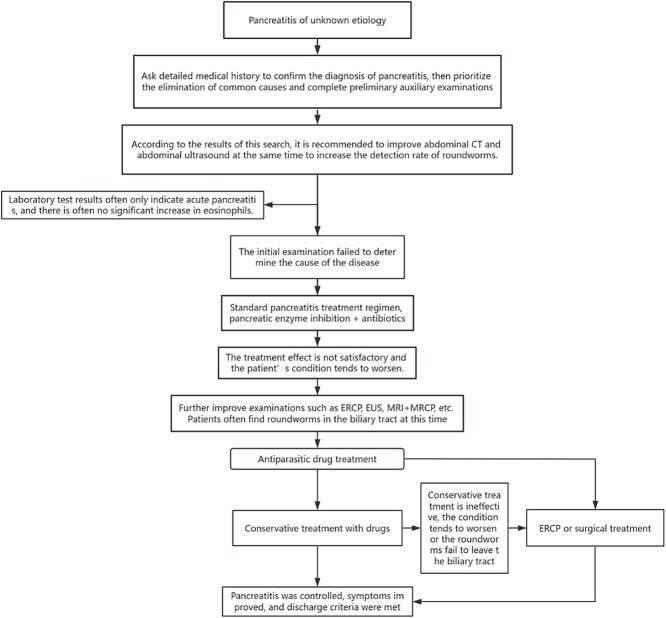
Diagnosis and treatment process after admission due to unclear initial diagnosis of ascariasis-induced pancreatitis.

This study found that most patients with Ascaris-induced pancreatitis were adults, and minors only accounted for a smaller proportion. According to Abhishek Mewar's description, this observation may be due to the smaller ampulla opening in children, which does not allow for the passage of roundworms [8]. Meanwhile, we found that there are more female cases than male cases. A previous study has suggested that this could be attributed to the relaxation effects of progesterone on the sphincter of Oddi in young women, making it easier for the roundworms to enter the bile duct. Progesterone inhibits smooth muscle contraction and has been shown to increase gallbladder volume while decreasing its emptying [9]. Secondly, clinical symptoms of acute pancreatitis caused by Ascaris worms are nearly similar to common conditions, such as biliary pancreatitis and hyperlipidemia pancreatitis. Only a few patients reported cases with the presence of roundworms in the vomitus. In such cases, the physician would consider ascaris worms as the possible cause. However, in most patients, standard treatment for acute pancreatitis is initiated upon admission. Thirdly, our results demonstrate that ultrasonic examination provides high sensitivity in the diagnosis of the disease. Nearly half of the patients were diagnosed with roundworm pancreatitis by ultrasonic examination. To avoid delay in treatment due to false negatives caused by CT analysis, an ultrasound examination is recommended. Notably, MRI + MRCP is superior to ERCP examination. Its non-invasive and non-radiation nature has attracted significant attention in recent times. Therefore, MRI + MRCP should be given priority as the core diagnostic tool. Finally, antiparasitic drugs should be the first choice in almost all patients, and additional treatment is determined based on the patient's response to treatment [10] (Fig. 4).

In cases where Ascaris worms do not leave the biliary tract or the condition worsens while receiving drug treatment, ERCP or surgical removal of the Ascaris worms is advised. This is due to the following reasons: 1. Anti-parasitism: The effective concentration of parasitic drugs that can enter the biliary tract after entering the blood is less than that of the gastrointestinal tract or other parts. 2. According to Mohammad S Khuro et al., if the Ascaris worms are still alive, they will repeatedly enter and exit the tubular cavity. Continuous ultrasound examination shows that the Ascaris worm activity in the tubular cavity usually lasts for ten days and may spontaneously exit the bile duct after that [11]. However, if anti-parasitic treatment has already been initiated, the Ascaris worms cannot leave the bile duct alone and are excreted through the gastrointestinal tract. Dead ascaris worms and ascaris worms eggs trapped in the bile duct will form and create lesions that lead to stone formation. This will chronically stimulate the bile duct and increase the risk of repeat infections and obstruction, a process in which *Escherichia coli* has been implicated [12]. Therefore, once the diagnosis of ascariasis-induced pancreatitis is made, conservative therapy should be initiated. Follow-up ultrasound examinations can be performed to monitor whether the ascaris lumbricoides have exited the bile duct.

However, for patients with biliary ascaris infection, if conservative treatment for 2–3 weeks is ineffective, the medical team usually adopts more aggressive intervention measures. The available invasive treatments mainly include ERCP and surgical procedures. A review of the results indicated that ERCP contributes to the treatment selection (85.45%). Natural cavity operation can intuitively locate and remove ascarides and timely perform biliary drainage. Small trauma and fast recovery are its advantages. The reason for choosing laparoscopic surgery, in this case, is that it still has irreplaceable advantages compared to ERCP: 1. If acute gallbladder inflammation or gallbladder ascaris lumbricoides are found during the operation, cholecystectomy can be performed simultaneously to avoid further surgery. 2. Even if ascarides are found in the intrahepatic bile duct or gallbladder during the operation, they can be easily removed or the surgical procedure can be flexibly changed to solve the problem. 3. Laparoscopic bile duct exploration can avoid incision or accidental damage to the Oddis sphincter muscle, reducing the risk of postoperative bile duct reflux or recurrent bile duct ascaris lumbricoides. 4 Since the development of laparoscopic technology, surgical time, intraoperative trauma, and postoperative recovery time have been greatly improved compared to before. Aligned with the principles of enhanced recovery after surgery, patients now experience significantly shorter hospital stays and a markedly improved overall treatment experience. Studies have shown that the length of hospital stay and hospitalization costs for laparoscopic primary closure of the common bile duct exploration (LCBDE-PC) are lower than those of the ERCP group [13]. Therefore, we believe that when selecting specific treatment plans, individualized assessments should be made based on imaging features, infection severity, and the patient's overall condition to develop personalized and optimal treatment plans.

## Conclusion

Ascariasis-induced pancreatitis is more common among Asians, being more prevalent in adult females, presenting with atypical symptoms compared to other pancreatitis patients. In cases of acute pancreatitis caused by biliary tract ascaris lumbricoides, it is recommended that clinical doctors use non-invasive tests such as serological tests, color ultrasound, and MRI + MRCP to assist in diagnosis. Although ERCP remains the most commonly reported treatment approach, laparoscopic surgery has increasingly demonstrated its benefits in recent years, particularly in terms of faster recovery. Its advantages become especially evident in complex cases, such as severe gallbladder inflammation or the presence of Ascaris lumbricoides in the intrahepatic bile ducts, where laparoscopy offers distinct and often irreplaceable benefits.

### Key clinical message

In cases of acute pancreatitis of unknown cause, further examination is necessary based on the patient's condition, including the possibility of ascariasis. A systematic assessment will help select appropriate treatment for optimal long-term outcomes.

## Data Availability

The datasets generated during and/or analyzed during the current study are available from the corresponding author upon reasonable request.
